# Efficacy and Outcomes of Intrathecal Analgesia as Part of an Enhanced Recovery Pathway in Colon and Rectal Surgical Patients

**DOI:** 10.1155/2018/8174579

**Published:** 2018-03-01

**Authors:** Amit Merchea, Jenna K. Lovely, Adam K. Jacob, Dorin T. Colibaseanu, Scott R. Kelley, Kellie L. Mathis, Grant M. Spears, Marianne Huebner, David W. Larson

**Affiliations:** ^1^Division of Colon and Rectal Surgery, Mayo Clinic, Jacksonville, FL, USA; ^2^Hospital Pharmacy Services, Mayo Clinic, Rochester, MN, USA; ^3^Department of Anesthesiology, Mayo Clinic, Rochester, MN, USA; ^4^Division of Colon and Rectal Surgery, Mayo Clinic, Rochester, MN, USA; ^5^Department of Biostatistics, University of Rochester, Rochester, MN, USA; ^6^Department of Statistics, Michigan State University, East Lansing, MI, USA

## Abstract

**Purpose:**

Multimodal analgesia is an essential component of an enhanced recovery pathway (ERP). An ERP that includes the use of single-injection intrathecal analgesia (IA) has been shown to decrease morbidity and cost and shorten length of stay (LOS). Limited data exist on safety, feasibility, and the optimal IA regimen. Our objective was to characterize the efficacy, safety, and feasibility of IA within an ERP in a cohort of colorectal surgical patients.

**Methods:**

We performed a retrospective review of all consecutive patients aged ≥ 18 years who underwent open or minimally invasive colorectal surgery from October 2012 to December 2013. All patients were enrolled in an institutional ERP that included the use of single-injection IA. Demographics, anesthetic management, efficacy (pain scores and opiate consumption), postoperative ileus (POI), adverse effects, and LOS are reported.

**Results:**

601 patients were identified. The majority received opioid-only IA (91%) rather than a multimodal regimen. Median LOS was 3 days. Overall rate of ileus was 16%. Median pain scores at 4, 8, 16, 24, and 48 hours were 3, 2, 3, 4, and 3, respectively. There was no difference in postoperative pain scores, LOS, or POI based on intrathecal medication or dose received. Overall, development of respiratory depression (0.2%) or pruritus (1.2%) was rare. One patient required blood patch for postdural headache.

**Conclusion:**

Intrathecal analgesia is safe, feasible, and efficacious in the setting of ERP for colorectal surgery. All regimens and doses achieved a short LOS, low pain scores, and a low incidence of POI. This trial is registered with Clinicaltrails.gov NCT03411109.

## 1. Introduction

Enhanced recovery pathways have been shown to decrease morbidity, length of hospital stay, and costs in colorectal surgery [1–6]. These pathways are designed to accelerate patient recovery and focus on certain key components—early feeding, maintenance of euvolemia, optimization of pain control (with limitation of systemic opioids), and early ambulation [5, 7–12]. Multimodal analgesia is an essential component of an enhanced recovery pathway (ERP). The use of continuous, low-thoracic epidural analgesia has long been cited as a beneficial and effective mode of analgesia in colorectal surgery [13]. The rationale for its use has been based on historical data that epidural analgesia provides more effective pain control and is associated with lower incidence of postoperative ileus after intra-abdominal surgery [14]. However, the use of epidural analgesia is not without limitations, including higher incidence of urinary retention, pruritus, hypotension, and potential lower extremity weakness preventing postoperative activity [14]. Recent publications have demonstrated the safety and efficacy of single-injection intrathecal analgesia (IA) for acute postoperative pain control in patients undergoing colorectal surgery [1, 15–18].

Long-acting opioids are considered the cornerstone of single-injection IA for postoperative pain. While morphine has been considered the gold standard opioid for IA, recent national drug shortages have limited its availability. Hydromorphone is an alternative long-acting opioid that has grown in popularity, particularly for chronic pain. Its role in acute postoperative pain is less clear. Limited data exist on the safety, efficacy, and optimal IA medication regimen in the setting of an ERP including open and laparoscopic surgery. Therefore, we aimed to describe the efficacy and safety of IA, including intrathecal hydromorphone, in a cohort of colon and rectal surgical patients in a standardized ERP program.

## 2. Materials and Methods

Institutional review board approval was obtained (ID # 13-007936). We retrospectively identified all colorectal procedures from October 2012 through December 2013 in which patients received single-injection IA as part of a multimodal analgesic strategy for ERP. Patients were identified from the prospectively-defined Mayo Clinic Colorectal Surgery Database. Inclusion criteria were age ≥ 18 years, undergoing an elective colorectal operation (minimally invasive or open), enrollment into the institutional perioperative ERP, and receiving preoperative single-injection IA. Patients undergoing emergent operations or who refused research authorization were excluded.

Our institution's enhanced recovery pathway has previously been described [6]. The analgesia regimen includes preoperative celecoxib, gabapentin, and acetaminophen, single-injection IA and additional intraoperative opiates (at the discretion of the anesthesiologist), scheduled nonsteroidal anti-inflammatory medications (NSAIDs), and acetaminophen postoperatively with additional oxycodone as needed. NSAIDs are excluded in patients with renal impairment (glomerular filtration rate < 50), with tramadol serving as the alternative. All intrathecal injections were performed preoperatively using a 22 g or 25 g Whitacre or 24 g Sprotte spinal needle. The IA regimen, medication(s) and dose(s), was at the discretion of the attending anesthesiologist and consisted of one of the following regimens [1]: hydromorphone + local anesthetic (IA-L) or [2] hydromorphone only (IA-O). In patients receiving IA, no other interventional locoregional analgesic techniques (such as rectus sheath blocks or transversus abdominis plane blocks) were utilized.

All patients were monitored according to institutional guidelines outlined in a standardized intrathecal for analgesia order set. This consists of continuous pulse oximetry and nursing assessments every hour for first 12 hours, then every 2 hours for next 12 hours, then every four hours in a general care setting. Intensive care unit monitoring was not routinely required [19].

Demographic data (age and sex) and operative details including anesthetic/analgesic management (dosage and pain scores) were collected from the anesthetic record. Short-term postoperative outcomes including verbal rating pain scores at 4, 8, 12, 24, and 48 hours after PACU discharge, postoperative opiate consumption (in oral morphine equivalents (OME)), postoperative ileus (defined as requirement of NGT postoperatively, inability to tolerate general diet by postoperative day 5, or clinician's documentation of ileus in postoperative course), and length of stay (LOS; prolonged length of stay was defined as >3 days) were collected from the colorectal surgical database. Adverse effects related to neuraxial opiate administration were recorded, specifically searching for the administration of naloxone as a surrogate metric for respiratory depression and nalbuphine as a surrogate for pruritus within 24 hours of surgery. Finally, presence of postdural puncture headache and requirement for a blood patch were collected using a free-text search of the medical record.

Continuous variables were summarized with mean and standard deviation (SD) or median and interquartile range (IQR). Categorical variables were summarized with frequencies and percentages. The comparison of continuous variables between groups was performed using the one-way ANOVA *F*-test or nonparametric Kruskal-Wallis test. For categorical variables, the chi-square or Fisher's exact test were used. A *p* value of 0.05 was considered statistically significant. All analyses were performed used SAS.

## 3. Results

A total of 601 patients were included in the analysis. Summary data are presented in Table 1. The majority of patients (*n*=547, 91%) received IA-O. There was no statistically significant difference observed in length of stay (LOS) when comparing IA-O or IA-L. Pain scores were also similar; however, the median 48 hour postoperative maximal pain score reported was higher in those receiving IA-L (7 versus 6, *p*=0.045). Furthermore, a greater proportion of those patients receiving IA-O utilized zero OMEs compared to IA-L (30 versus 15%, *p*=0.03) (Table 2).

Patients were grouped into one of four hydromorphone dose ranges: <50 mcg, 50–75 mcg, 76–100 mcg, and >100 mcg (Table 3). The majority of patients received 76–100 mcg of intrathecal opioid (*n*=427, 71%). There was no difference in the reported pain scores at all measured intervals. However, the total oral morphine equivalents (OME) utilized were greater in those patients with higher dose IA, specifically those receiving 76–100 mcg or >100 mcg (*p*=0.01). When eliminating those patients who received zero OMEs, there was no difference in OME received between the dose ranges (*p*=0.14). Fifty-five patients received >200 OMEs; when reviewing these patients, the majority (*n*=42) had either an open operation with midline incision or a hand-assisted approach.

Median (IQR) length of stay (LOS) for the entire cohort was 3 days [2, 5]. LOS did not differ between those receiving IA-O and IA-L (median 3 days versus 3.5 day, *p*=0.29). The median (IQR) LOS in patients with a LOS greater than 3 days (*n*=258, 43%) was 5 days [4, 8]; ileus was noted in 36% of these patients with an LOS greater than 3 days. The median maximal pain score in the 48 hours after surgery was 5 in those with a LOS < 3 days versus 7 in those patients with a LOS > 3 days (*p* < 0.0001).

Complications of IA were rare. Seven patients (1.2%) were treated with nalbuphine for symptoms of pruritus, and one patient (0.2%) developed respiratory depression requiring administration of naloxone within 24 hours of the administration of IA. One patient (0.2%) required blood patch for postdural puncture headache.

## 4. Discussion

Our review of the feasibility, safety, and efficacy of intrathecal analgesia in a high-volume enhanced recovery colorectal surgery program demonstrated that intrathecal analgesia is feasible, safe, and effective across a wide range of opioid dosages. Furthermore, lower dosages of intrathecal opioid appeared to achieve a similar level of pain control.

Although the beneficial effects of epidural analgesia have been widely reported [20–24], the use of single-dose intrathecal injection is gaining favor as increasing reports of its safety and efficacy are published [1, 15, 17]. The single injection affords a feasibility advantage as no additional equipment (cost) or ‘tether' (ongoing catheter) is required. In our institution, an anesthesiologist-based pain service provides on-call support for 24 hours after the injection, whereas they would be consulted and actively managed for 2-3 days duration if an epidural option were utilized. This decreased use of intense resources and increased feasibility for implementation in our institution may ultimately apply to other settings. There has been a historical perception that intrathecal is more hazardous; however, many authors have demonstrated otherwise, along with earlier return of bowel function, earlier ambulation, and shorter length of stay [13, 15–17]. To our knowledge, our report is the first large-volume single-institution review of patients undergoing intrathecal analgesia in the setting of an established colorectal surgery ERP and the first within ERP to specifically examine the effect of various intrathecal-dosing regimens.

Among the chief concerns often cited with the use of long-acting intrathecal opiates is the risk of respiratory depression. Our report demonstrated this to be a rare event (0.2%). This is less than other contemporary studies, with reported incidences as high as 3% [15, 18, 25]. Routine intensive postoperative monitoring in a higher level of care (e.g., ICU) is not required. The standard monitoring of pulse oximetry and nursing assessments every hour for first 12 hours, then every 2 hours for next 12 hours, then every four hours in a general care setting is likely adequate [19]. However, one must identify those patients who may be at higher risk of developing respiratory depression—those who are morbidly obese, with obstructive sleep apnea, and those with previous adverse respiratory events associated with opioids [19]. Furthermore, patients rarely experience spinal headache or pruritus—only one patient in our series required a blood patch for spinal headache. The previously reported incidence of postdural puncture headache is 0–13% [26]. To further lessen each of these risks, lower doses of intrathecal hydromorphone (50 mcg) may be used and may still achieve similar rates of pain control and LOS outcomes. This will be incorporated into the collective knowledge for this practice for care of future patients.

Intrathecal analgesia within an ERP is performed to provide an additional pain control mechanism within a multimodal pathway that also includes acetaminophen (paracetamol) and NSAIDs. Larson et al. previously reported our experience that pathway compliance, low postoperative oral morphine equivalent (OME) usage (<30 mg), and high surgeon volume (>100 cases per year) were associated with discharge from the hospital within 48 hours [27]. In this series, Larson et al. described an 82.4–99.3% compliance rate of seven measured elements of our ERP pathway. Specifically, for intrathecal analgesia, the compliance rate was 84%. We believe that intrathecal is a critical element that aids in achieving the low OME goal being met and thus facilitates early discharge. In our present review, median LOS was 3 days, and this was consistent across all dose ranges of intrathecal. Furthermore, reported pain scores were equivalent in all groups. In our review, the patients with the highest IA doses utilized the highest OMEs. When eliminating those with highest OMEs, there was no statistical difference in the OMEs received in all dose ranges. This suggests that high OME usage is independent of IA dose.

Consistent with best practices for anesthesiology and pharmacists, we generally feel that the lowest effective dose should typically be employed. Though limited data are available on the use of intrathecal hydromorphone for acute postoperative pain, a recent prospective comparison of spinal hydromorphone and morphine in cesarean section determined that the ED90 of intrathecal hydromorphone was 75 mcg [28]. Our results would corroborate these findings; patients receiving 50–75 mcg appeared to have similar analgesic outcomes compared to patients who received >75 mcg.

The major limitation of this study is that it was conducted as a retrospective cohort study, which in itself presents inherent weaknesses such as incomplete documentation or subjective, biased reporting of outcomes. Though premedication and postoperative analgesic protocols were identical, group assignment to treatment with intrathecal hydromorphone was not randomized or blinded, and the dose of hydromorphone was based on the preference of the attending anesthesiologist. Therefore, potential for selection bias exists with hydromorphone dose and older age and/or type of procedure (open versus MIS). Randomized trials further investigating alternative dosing regimens would limit the inherent associated biases. However, we are still able to report a relatively robust experience and characterize the feasibility, safety, and efficacy of IA.

## 5. Conclusion

In conclusion, IA is a safe and efficacious mode of analgesia for patients undergoing open and minimally invasive colorectal surgery. All intrathecal regimens and doses were efficacious at achieving a short LOS, low pain scores, and a low incidence of adverse events.

## Figures and Tables

**Table 1:** Summary data.

Age at operation, median (IQR)	52 (37–65)
Patient sex, *n* (%)	
Male	308 (51)
Female	293 (49)
Surgical approach, *n* (%)	
Open	356 (59%)
Minimally invasive	245 (41%)
Length of stay (days), median (IQR)	3 (2–5)
Intrathecal medications administered, *n* (%)	
Opioid only	547 (91%)
Opioid + local anesthetic	54 (9%)
Ileus, *n* (%)	95 (16%)
Postoperative pain scores, median (IQR)	
4 hours	3 (1–5)
8 hours	2 (1–4)
16 hours	3 (1–5)
24 hours	4 (2–5)
48 hours	3 (2–5)
48-hour maximum pain score, median (IQR)	6 (4–7)
Total OME, median (IQR)	24 (0–83)
Number of patients receiving no OME, *n* (%)	170 (28%)

OME: oral morphine equivalent; IQR: interquartile range.

**Table 2:** Comparison of various intrathecal regimens.

	IA-O (*n*=547)	IA-L (*n*=54)	*p* value
Surgical approach, *n* (%)			0.15
Open	329 (60)	27 (50)	—
MIS	218 (40)	27 (50)	—
Incidence of ileus, *n* (%)	86 (16)	9 (17)	0.86
Length of stay (days), median (IQR)	3 (2–5)	3.5 (3–5)	0.29
Pain score, median (IQR)			
4 hours	3 (1–5)	3 (2–5)	0.52
8 hours	2 (1–4)	2.5 (2–4)	0.16
16 hours	3 (1–5)	4 (2–7)	0.10
24 hours	3 (2–5)	4 (2–6)	0.32
48 hours	3 (2–5)	4 (2–5)	0.12
48-hour maximum	6 (4–7)	7 (5–8)	**0.045**
Total OME received, median (IQR)	22.5 (0–82.5)	33.8 (15–98)	0.06
Number of patients receiving no OME, *n* (%)	162 (30)	8 (15)	**0.03**

IA: intrathecal opioid; IA-L: intrathecal opioid + local anesthetic; MIS: minimally invasive surgery; IQR: interquartile range.

**Table 3:** Comparison of opioid dosing regimens.

	<50 mcg (*n*=20)	51–75 mcg (*n*=36)	76–100 mcg (*n*=427)	>100 mcg (*n*=118)	*p* value
Type of intrathecal, *n* (%)					0.38
IA-O	20 (100)	34 (94)	388 (91)	105 (89)	—
IA-L	0 (0)	2 (6)	39 (9)	13 (11)	—
Incidence of ileus, *n* (%)	5 (25)	2 (5.6)	70 (16)	18 (15)	0.24
Length of stay (days), median (IQR)	4 (2–5.5)	3 (2–4.5)	3 (2–5)	3 (2–5)	0.58
Pain score, median (IQR)					
4 hours	2 (0.5–3)	3 (1–4)	3 (2–5)	3 (1–4)	0.30
8 hours	2 (0–3)	2 (1–3)	2 (1–4)	2 (1–4)	0.76
16 hours	3.5 (2–5)	3 (2–5)	3 (2–5)	2 (1–5)	0.42
24 hours	3 (1–4)	2.5 (2–4.5)	4 (2–5)	3 (2–6)	0.25
48 hours	2 (1–3)	3 (2–4)	3 (2–5)	3 (2–6)	0.29
48-hour maximum	5 (4–7.5)	5 (4–7)	6 (4–7)	6 (4–8)	0.52
Total OME used, median (IQR)	7.5 (0–37.5)	7.5 (0–48.8)	30 (0–90)	22.5 (0–82.5)	**0.01**

IA: intrathecal opioid; IA-L: intrathecal opioid + local anesthetic; IQR: interquartile range.
